# The World Trade Center Disaster and the Health of Workers: Five-Year Assessment of a Unique Medical Screening Program

**DOI:** 10.1289/ehp.9592

**Published:** 2006-09-06

**Authors:** Robin Herbert, Jacqueline Moline, Gwen Skloot, Kristina Metzger, Sherry Baron, Benjamin Luft, Steven Markowitz, Iris Udasin, Denise Harrison, Diane Stein, Andrew Todd, Paul Enright, Jeanne Mager Stellman, Philip J. Landrigan, Stephen M. Levin

**Affiliations:** 1 Department of Community and Preventive Medicine and; 2 Division of Pulmonary, Critical Care & Sleep Medicine, Mount Sinai School of Medicine, New York, New York, USA; 3 Division of Surveillance, Hazard Evaluations, and Field Studies, National Institute for Occupational Safety and Health, Centers for Disease Control and Prevention, Cincinnati, Ohio, USA; 4 Department of Medicine, State University of New York at Stony Brook, Port Jefferson, New York, USA; 5 Center for Biology of Natural Systems, Queens College, Flushing, New York, USA; 6 Environmental and Occupational Health Sciences Institute, University of Medicine & Dentistry of New Jersey, Piscataway, New Jersey, USA; 7 Department of Environmental Medicine, Bellevue Hospital Center/New York University School of Medicine, New York, New York, USA; 8 Division of Respiratory Disease Studies, National Institute for Occupational Safety and Health, Centers for Disease Control and Prevention, Morgantown, West Virginia, USA; 9 Mailman School of Public Health, Columbia University, New York, New York, USA

**Keywords:** air pollution, disaster response, occupational lung disease, pulmonary function, September 11, spirometry, World Trade Center

## Abstract

**Background:**

Approximately 40,000 rescue and recovery workers were exposed to caustic dust and toxic pollutants following the 11 September 2001 attacks on the World Trade Center (WTC). These workers included traditional first responders, such as firefighters and police, and a diverse population of construction, utility, and public sector workers.

**Methods:**

To characterize WTC-related health effects, the WTC Worker and Volunteer Medical Screening Program was established. This multicenter clinical program provides free standardized examinations to responders. Examinations include medical, mental health, and exposure assessment questionnaires; physical examinations; spirometry; and chest X rays.

**Results:**

Of 9,442 responders examined between July 2002 and April 2004, 69% reported new or worsened respiratory symptoms while performing WTC work. Symptoms persisted to the time of examination in 59% of these workers. Among those who had been asymptomatic before September 11, 61% developed respiratory symptoms while performing WTC work. Twenty-eight percent had abnormal spirometry; forced vital capacity (FVC) was low in 21%; and obstruction was present in 5%. Among nonsmokers, 27% had abnormal spirometry compared with 13% in the general U.S. population. Prevalence of low FVC among nonsmokers was 5-fold greater than in the U.S. population (20% vs. 4%). Respiratory symptoms and spirometry abnormalities were significantly associated with early arrival at the site.

**Conclusion:**

WTC responders had exposure-related increases in respiratory symptoms and pulmonary function test abnormalities that persisted up to 2.5 years after the attacks. Long-term medical monitoring is required to track persistence of these abnormalities and identify late effects, including possible malignancies. Lessons learned should guide future responses to civil disasters.

An estimated 40,000 men and women worked at Ground Zero, the former site of the World Trade Center (WTC) in New York City, and at the Staten Island landfill, the principal wreckage depository in the days, weeks, and months after 11 September 2001 (Levin et al. 2004). These workers and volunteers included traditional first responders such as firefighters, law enforcement officers, and paramedics, as well as a diverse population of operating engineers, laborers, ironworkers, railway tunnel cleaners, telecommunications workers, sanitation workers, and staff of the Office of the Chief Medical Examiner. These men and women carried out rescue-and-recovery operations, restored essential services, cleaned up massive amounts of debris, and in a time period far shorter than anticipated, deconstructed and removed remains of buildings. Many had no training in response to civil disaster. The highly diverse nature of this work-force posed unprecedented challenges for worker protection and medical follow-up.

Workers were exposed to a complex mix of toxic chemicals and to extreme psychological trauma. These exposures varied over time and by location (Landrigan et al. 2004; Lioy et al. 2002). Combustion of 90,000 L of jet fuel immediately after the attacks created a dense plume of black smoke containing volatile organic compounds (including benzene), metals, and polycyclic aromatic hydrocarbons. The collapse of the twin towers (WTC 1 and WTC 2) and then of a third building (WTC 7) produced an enormous dust cloud containing thousands of tons of coarse and fine particulate matter (PM), cement dust, glass fibers, asbestos, lead, hydrochloric acid, polychlorinated biphenyls (PCBs), organochlorine pesticides, and polychlorinated dioxins and furans (Clark et al. 2003; Landrigan et al. 2004; Lioy et al. 2002; McGee et al. 2003). U.S. Environmental Protection Agency (EPA) estimates of airborne dust ranged from 1,000 to > 100,000 μg/m^3^ (U.S. EPA 2002). The high content of pulverized cement made the dust highly caustic (pH 10–11) (Landrigan et al. 2004; Lioy et al. 2002).

Dust and debris gradually settled, and rains on 14 September further diminished the intensity of outdoor ambient dust exposure. However, rubble-removal operations repeatedly reaerosolized the dust, leading to continuing intermittent exposure for many months. Fires burned both above and below ground until December 2001 (Banauch et al. 2003; Chen and Thurston 2002; U.S. EPA 2003). Air levels of certain contaminants remained elevated well into 2002, with spikes in benzene and asbestos levels occurring as late as March and May 2002, respectively (U.S. EPA 2003).

Workers began noting symptoms soon after September 11, most commonly involving the aerodigestive tract (upper and lower respiratory tract and esophagus) (Banauch et al. 2006; Salzman et al. 2004; Szeinuk et al. 2003). New York City Fire Department (FDNY) firefighters experienced persistent cough, termed the “World Trade Center cough,” which was accompanied by respiratory distress and bronchial hyperreactivity (Prezant et al. 2002). A sample of FDNY firefighters who had sustained extreme exposures on September 11 was nearly 8 times more likely to manifest bronchial hyperreactivity than firefighters with lower exposures when examined after 6 months (Banauch et al. 2003). Laborers and ironworkers manifested new-onset cough, wheeze, and sputum production (Geyh et al. 2005; Skloot et al. 2004), likely attributable to respiratory inflammation caused by the highly alkaline dust (Chen and Thurston 2002).

Other reported pulmonary effects included cough, asthma, and reactive airway dysfunction syndrome (Balmes 2006; Banauch et al. 2006). Chronic rhinosinusitis, vocal cord inflammation, and laryngitis (de la Hoz et al. 2004) and case reports of eosinophilic pneumonia (Rom et al. 2002), granulomatous pneumonia, and bronchiolitis obliterans (Mann et al. 2005; Safirstein et al. 2003) were also reported.

Although New York has an extensive hospital network and strong public health system, no existing infrastructure was sufficient for providing unified and appropriate occupational health screening and treatment in the aftermath of September 11. Local labor unions, who made up the majority of responders, became increasingly aware that their members were developing respiratory and psychological problems; they initiated a campaign to educate local elected officials about the importance of establishing an occupational health screening program. In early 2002, Congress directed the Centers for Disease Control and Prevention (CDC) to fund most of the WTC Worker and Volunteer Medical Screening Program (MSP), an action largely attributable to the collaborative efforts of organized labor and elected officials. The goals of the program were as follows:

- To rapidly build a regional and national consortium of occupational medicine clinics to conduct geographically convenient standardized medical evaluations
- To identify WTC responders, notify them about this program, and encourage participation
- To provide clinical examinations for eligible individuals to identify WTC-related physical and/or mental health conditions
- To coordinate referral for follow-up clinical care for affected individuals
- To educate workers and volunteers about exposures and associated risks to their health
- To advise affected individuals about available benefit and entitlement programs
- To establish “baseline” clinical status for individuals exposed at or near Ground Zero for comparison with future clinical assessments.

In April 2002, the Irving J. Selikoff Center for Occupational and Environmental Medicine (COEM) at Mount Sinai was awarded a contract by the National Institute for Occupational Safety and Health (NIOSH) to establish and coordinate the MSP. The Bellevue/New York University Occupational and Environmental Medicine Clinic, the State University of New York Stony Brook/Long Island Occupational and Environmental Health Center, the Center for the Biology of Natural Systems at Queens College in New York, and the Clinical Center of the Environmental & Occupational Health Sciences Institute at UMDNJ-Robert Wood Johnson Medical School in New Jersey were designated as the other members of the regional consortium. The Association of Occupational and Environmental Clinics was designated to coordinate a national examination program for responders who did not live in the New York/New Jersey area.

In this article we describe the design and implementation of the MSP and the prevalence of selected clinical findings from screening examinations conducted between July 2002 and April 2004 in those from whom informed consent and HIPAA (Health Insurance Portability and Accountability Act 1996) authorization were obtained. Mental health service provision and findings will be presented in a separate paper.

## Materials and Methods

### Establishing the cohort: identification and outreach

The target population was approximately 18,000 WTC responders not eligible to participate in other federally funded programs (e.g., FDNY, federal workers, New York State workers). Because responders came from many sectors, a high proportion as unpaid volunteers, no systematic roster of names and contact information was available. An MSP outreach unit was therefore established and staffed by people experienced in occupational health and familiar with key organizations, primarily labor unions representing responders.

### The MSP executive steering committee

To ensure key stakeholder input into all aspects of program development and oversight, an executive steering committee (ESC) was established; the ESC included representatives from each of the consortium clinics, representatives from labor unions, employers, and technical experts from relevant fields.

The ESC advised the program directors on all program decisions and on basic components of the medical examination, eligibility criteria, and the outreach plan. An advisory council of > 100 people was created several months after the start of the program to broaden stakeholder involvement and to tap into the enthusiasm and creativity of responder organizations. Generally 40–50 responder representatives attended quarterly advisory council meetings. The ESC and advisory council helped maintain open lines of communication with representatives of the program’s diverse responder population.

### Examination eligibility

To be eligible to receive an examination, a responder must have fallen into one of two categories: For the first category, the responder must have been a rescue, recovery, debris-cleanup and related support services worker, or volunteer in *a*) lower Manhattan (south of Canal St.), *b*) the Staten Island Landfill, and/or *c*) the barge loading piers, and must have worked and/or volunteered on-site for 4 hr on 11–14 September 2001, for at least 24 hr during the month of September, or for at least 80 hr during the months of September, October, November, and December combined.

To fall into the second category, the responder must have been an employee of the Office of the Chief Medical Examiner (OCME), involved in the examination and processing of human remains, or other morgue worker who performed similar post-September 11 functions for OCME staff; a worker in the Port Authority Trans-Hudson Corporation tunnel through 1 July 2002 for a minimum of 24 hr; or a vehicle-maintenance worker with post-September 11 functions within the requisite timeframes and exposed to WTC debris while retrieving, driving, cleaning, repairing, and maintaining contaminated vehicles.

### Development of the examination protocol

The clinical consortium partners, supplemented by experts in psychiatry, pulmonary medicine, otolaryngology, industrial hygiene, and epidemiology, collaborated in protocol development to provide high quality standardized occupational health screening examinations and gather information for a research database to enable scientific assessment of the full health impact of the disaster. Early in protocol planning it was decided that direct clinical services had priority where clinical protocols conflicted with collection of research data.

### Standardized medical examination

Responders received a clinical screening evaluation consisting of medical, mental health, and exposure-assessment questionnaires; a standardized physical examination; and pre- and postbronchodilator spirometry, complete blood count, blood chemistries, urinalysis, and chest radiograph. Participants received both immediate and final letters with examination results and a face-to-face physician consultation at the end of the examination day. Participants were provided referrals for evaluation and treatment for physical or mental health conditions identified in the screening examination.

A trained health care practitioner administered a medical questionnaire on selected diagnoses and prior upper and lower respiratory conditions (e.g., chronic sinusitis and asthma), occurrence of symptoms in the year before 11 September 2001, during the period the subject worked at the WTC site, for the month before the screening examination, and whether preexisting symptoms and diagnoses worsened during their WTC work. A questionnaire also asked about smoking history. Where possible, questions were adapted from standardized instruments (e.g., Burney et al. 1989; European Community Respiratory Health Survey 1994; Miller et al. 2005; National Center for Health Statistics 1996; NIOSH 2006; Piccirillo et al. 2002).

We used an interviewer-administered survey instrument to obtain pre- and post-September 11 occupational and environmental exposure histories, including dates that responders reported for first working or volunteering for September 11–related duties and, for those present on September 11, whether they were exposed to the cloud of dust from the building collapses. We constructed the ordinal date-related categories shown in the tables as a rough measure of relative dust exposures, and also categorized workers by location where they spent the majority of their time when first working at Ground Zero. We also obtained data on respirator type and use during the first week of the WTC recovery; those data will be reported in subsequent analyses.

Eligible responders were invited for clinical examinations irrespective of their willingness to provide consent to have data aggregated. Only data from responders providing institutional review board consent and HIPAA authorization (on or after 14 April 2003) are included in data analyses.

### Spirometry

Spirometric examination employed the EasyOne spirometer (ndd Medical Technologies, Chelmsford, MA) using standard techniques (Miller et al. 2005). We compared spirometry results to age-, sex-, and ethnic-specific reference values derived from the third phase of the National Health and Nutrition Examination Survey (NHANES III) (Hankinson et al. 1999). Interpretation followed the recently combined American Thoracic Society and European Respiratory Society guidelines (Pellegrino et al. 2005). Only spirometry of acceptable quality, as defined by international guidelines (Miller et al. 2005), was included in the analysis (*n* = 8,384). Airway obstruction was defined as forced expiratory volume/forced vital capacity (FEV_1_/FVC) below the lower limit of normal (LLN) with a normal FVC. Spirometry with FVC < LLN but FEV_1_/FVC ≥ LLN was categorized as “low FVC.” Obstruction and low FVC was defined as FEV_1_/FVC < LLN and FVC < LLN. A significant bronchodilator response was defined as an increase in FEV_1_ or FVC of < 12% and 200 mL. Comprehensive spirometry quality assurance was an integral aspect of this program.

### Data analysis

We used SAS software (version 9.1; SAS Institute, Inc., Cary, NC) for all analyses. Categorization of occupational sector was based on the union and/or organization to which the responder reported belonging during work on the WTC effort. We categorized prevalence of specific health outcomes by date of arrival and exposure to the dust cloud and used the Cochrane-Armitage trend test to assess significance of trends in prevalence across exposure categories.

## Results

The MSP began examining responders in July 2002, 3 months after receipt of federal funding. Of the 16,528 responders meeting eligibility criteria, we examined 11,095 responders in the New York/New Jersey regional clinical consortium and 645 elsewhere between 16 July 2002 and 16 April 2004. In the New York/New Jersey consortium, 9,442 of these responders provided appropriate consent to be included in this report.

### Demographics

The responders screened in this program were predominantly male (87%) and white (66%), with a median age of 42 years (range, 18–82 years) (Table 1). More than 92% lived in the tristate (New York, New Jersey, Connecticut) area, 54% from New York City and 15% on Long Island; 86% were union members; 34% were construction workers; and 29% worked in law enforcement. We conducted > 14% of the examinations in languages other than English.

### Time of arrival and location

Of the > 40% of the responders who first arrived for work at the site on September 11, 49% reported having been engulfed in the building-collapse dust cloud (Table 1). Another 30% first arrived on 12 or 13 September. Irrespective of date of arrival, 35% of responders began working on the pile or in the pit at Ground Zero; another 55% worked adjacent to the pile; and the remaining 10% worked at other sites. The reported average duration of exposure (the time between the first and last days of work on the WTC effort) was 171 days (range, 1 day to ≥ 2.5 years). The average time between first work day and the MSP examination was 20 months.

### Symptoms

Most of the 9,442 responders examined reported being asymptomatic in the year prior to September 11 for lower respiratory tract symptoms (85%), and a large majority (66%) were asymptomatic for upper respiratory tract symptoms (Table 2). In the previously asymptomatic group, 44% reported developing lower respiratory symptoms and 55% developed upper respiratory symptoms while engaged in WTC-related work. These new symptoms were persistent in many; at the time of exam, 32% reported current lower respiratory symptoms and 44% reported current upper respiratory symptoms (Table 2). Fully 69% of all responders reported having had at least one worsened or newly incident respiratory symptom while performing WTC response work (63% upper airway and 47% lower airway symptoms, with overlap between the groups) (Table 3). Respiratory symptoms persisted to the time of examination in 59% of the population.

Early arrival at the WTC site was significantly associated with an increased reported prevalence of both newly incident and worsened respiratory symptoms (Table 3). We observed the highest prevalence among those who arrived on September 11 and were exposed to the dust cloud (54% lower respiratory and 66% upper respiratory symptoms). Those who began work on September 11 but who were not directly exposed to the dust cloud had the next highest prevalence (47% lower respiratory and 62% upper respiratory symptoms). We found a continuing statistically significant downward trend (although the prevalence remained high) in the incidence of reported symptoms for later arrival dates. Even those responders who arrived at the site on or after 1 October had a 41% prevalence of lower respiratory and a 59% prevalence of upper respiratory symptoms, nearly three times the percentage who had reported lower respiratory symptoms in the year prior to September 11, and nearly twice of the percentage who reported prior upper respiratory symptoms.

Of the 8,384 participants with acceptable quality pulmonary function exams, 28% had abnormal prebronchodilator spirometry results (Table 4). A low FVC was the most common abnormality (21%), whereas obstruction occurred in 5% and a mixed pattern (obstruction and low FVC) in 2%. We also documented a significant response to bronchodilator in 910 (11%) of participants including 33% of those with obstruction, 56% with a mixed pattern, and 18% of those with a low FVC.

Compared with a U.S. general population sample of employed, adult, white males (Mannino et al. 2003), the 4,641 participants who had never smoked had a higher prevalence of abnormalities on spirometry (27% vs. 13%). The difference was mainly attributable to a higher prevalence of tests with a low FVC (20% vs. 4%).

We observed a statistically significant association between time of arrival and low FVC, with a higher prevalence of abnormality in those who arrived earlier (Table 5). There was no significant difference in the prevalence of obstruction based on onset of exposure.

Thirty-one percent of the sample reported having received medical care for WTC-related respiratory conditions. A total of 17% of examinees reported missing work because of WTC-related health problems. Of the 1,973 workers with a self-reported diagnosis of sinusitis, 40% were seen by a doctor for this condition during the 6 months after September 11, compared to only 13% in the 6 months before September 11. Similar increases were reported in the numbers of responders who sought medical help for acute bronchitis (45% vs. 18%) and pneumonia (10% vs. 1%).

## Discussion

Two principal lessons emerge from our experiences with the WTC MSP. First, the prevalence rates of respiratory and other symptoms, and the prevalence of pulmonary function abnormalities in the nearly 10,000 WTC workers and volunteers whom we examined clinically between 2002 and 2004 were very high, and they are persistent. Health effects were most frequent in responders who sustained the most intense exposures. In the aftermath of future civil disasters, hospitals and health care providers will need to anticipate and prepare for the severe health consequences that inevitably result from the extreme exposures sustained by workers in these situations.

Second, in the event of future disasters, it is likely that existing health care facilities and public health programs will not be sufficiently robust or flexible to deal with the special needs and complex health problems sustained by responders and victims. It will likely be necessary to establish large, multicenter medical follow-up programs such as were needed in New York. The more rapidly such programs can be established and funded, the more quickly essential services will be provided (Rosner and Markowitz 2006).

Abnormal spirometry was still evident in almost one-third of all WTC workers and volunteers 1–2.5 years after 11 September 2001. The most common spirometric abnormality seen was a low FVC, which had also been found in the first 1,138 participants from this group (Levin et al. 2004). Low FVC was about 5 times more prevalent among nonsmokers than expected in the general U.S. population, based on NHANES III data (Mannino et al. 2003). Prevalence of low FVC was higher in responders who arrived at the disaster site closer to the time of the collapse of the twin towers than in those who arrived on or after 1 October.

There are several possible explanations for the high rates of low FVC observed in this group: *a*) true restriction due to parenchymal lung disease (e.g., interstitial lung diseases such as sarcoidosis, idiopathic pulmonary fibrosis, pneumoconiosis); *b*) true restriction due to physical factors such as obesity or chest wall abnormalities; *c*) “pseudorestriction” due to air trapping (e.g., airways obstruction) or sub-maximal inspiratory and/or expiratory effort (typically the result of chest pain/tightness or in an attempt to reduce coughing during the test); or *d*) our selection of the reference value used to define the lower limit of the normal range for FVC.

It is likely that, in some responders, the observed increase in low FVC is due to air trapping in the lungs, possibly due to inhalation of caustic dust and airborne pollutants in the course of their WTC work. A finding that supports this explanation is our observation of an increase in FVC after administration of a bronchodilator, seen in 18% of WTC workers and volunteers with this pattern.

Another possible explanation for our observed abnormalities in pulmonary function is our choice for the lower reference limit of the normal range for FVC. In our analysis we chose to use the Hankinson pulmonary reference values derived from NHANES III (Hankinson et al. 1999), because we considered them to be most appropriate for an ethnically diverse population such as this workforce. In previous studies of workers, several spirometry reference equations other than those from NHANES III have been used (Crapo et al. 1981; Knudson et al. 1983; Miller et al. 1983; Morris et al. 1973). Although the mean predicted values calculated from these five studies are very similar for whites, differences in the lower limits of the normal range provide large differences in spirometry abnormality rates when testing large, ethnically diverse groups of workers. For example, when using the equations from Crapo et al. (1981), substantially higher rates of obstruction but lower rates of spirometric restriction (low FVC) were found in whites in our cohort. It is also possible that some responders have developed true restrictive lung disease due to their WTC-related exposures. We anticipate that these issues will become clearer with continuing prospective follow-up of this cohort.

The MSP faced many challenges, and similar challenges are likely to arise in future major civil disasters. We faced organizational challenges in coordinating work at five clinical sites in the New York/New Jersey metropolitan area, as well as in the national program. There was no systematic roster of responders. We found that a broad and vigorous outreach program to systematically identify responders and persuade them of the importance of undergoing examination was essential. Most of these workers, many of whom had volunteered their services after September 11, were unable to take paid time off to be screened, and many were not in the position to forfeit a day’s wages. We needed to schedule the examinations at times and in locations that respected those difficulties. The examination content needed to be relevant and acceptable to the responders and at the same time sufficiently standardized to permit interpretation of aggregated clinical data. Translation was one of the more challenging aspects of program coordination. More than 14% of responders required non-English examinations and written materials.

The need for follow-up medical treatment and for provision of social benefits in the event of future civil disasters must be anticipated, and federal funds must be provided early on to support such programs. There was substantial social and economic disruption to the lives of many of the responders, and benefits counseling became an urgent need and an integrated component of the MSP. Many responders needed follow-up treatment for physical or mental health illnesses, and many lacked health insurance. We were obliged to secure private funding from philanthropic organizations to develop and implement treatment programs for responders. Federal funding for treatment of these workers is anticipated to begin in fall 2006.

Several limitations in these data should be noted. We do not have pre-September 11 clinical information on our cohort. It may be that responders who were sicker were more likely to participate, leading to an overestimation of risk. Conversely, we may be underestimating risk because most responders were likely to have been fit workers (healthy worker effect). In this article we do not consider the psychological consequences, which we already know to be serious (Smith et al. 2004). Subsequent papers will address responder mental health.

## Conclusions

The workers and volunteers who served New York City and the nation through their heroic service in the aftermath of September 11 need continuing medical surveillance and follow-up, especially because some diseases, such as cancer, are of long latency. Malignant mesothelioma resulting from exposure to asbestos, for example, may not become evident for 30–50 years. These biological facts plus the magnitude and complexity of the exposures indicate that WTC responders should be monitored for at least 20–30 years, so that long-term effects are detected early, when treatment would be most beneficial.

Federal leadership is needed to bring together a wide range of civilian and military experts to prepare for the complex physical and mental health issues and the environmental issues certain to arise in future disasters. Future disaster response must incorporate rapid establishment of both diagnostic and treatment programs, and state and federal leadership must make a firm commitment for the long-term follow up of exposed workers. Finally, there is a need to ensure strong and active participation by worker representatives and local citizens. Their local knowledge is unique, and it will not become available to state and federal planners unless these vital stakeholders are invited to take an active role in the planning and implementation of responses to future disasters.

## Figures and Tables

**Table 1:** Demographic and exposure characteristics of the WTC MSP study population (*n* = 9,442).

	No. (%)
Sex	
Male	8,186 (86.7)
Female	1,256 (13.3)
Race	
White	6,203 (65.7)
Black	1,060 (11.2)
Asian	121 (1.3)
Other	253 (2.7)
Unknown	1,805 (19.1)
Hispanic ethnicity	
Yes	2,249 (23.8)
Language of exam	
English	8,114 (85.9)
Spanish	984 (10.4)
Polish	311 (3.3)
Other	33 (0.3)
Union member	
Yes	8,075 (86.0)
Union/organization affiliation	
Construction	3,209 (34.0)
Law enforcement	2,776 (29.4)
Public sector (blue collar)	739 (7.8)
Technical and utilities	683 (7.2)
Transportation	516 (5.5)
Cleaning/maintenance	258 (2.7)
Volunteers	245 (2.6)
Firefighters^a^	138 (1.5)
Health care	83 (0.9)
News agencies	81 (0.9)
Office/administration/professional	50 (0.5)
Other	664 (7.0)
Time first began WTC-related work	
11 September 2001	3,812 (40.5)
In dust cloud	1,878 (20.0)
Not in dust cloud	1,934 (20.5)
12–13 September 2001	2,801 (29.8)
14–30 September 2001	2,133 (22.7)
On or after 1 October 2001	666 (7.1)
Location of majority of work	
On the pile/in the pit	3,215 (34.8)
Adjacent to pile/pit	5,074 (54.8)
Landfill	313 (3.4)
Barges/loading pier	106 (1.1)
OCME	77 (0.8)
Elsewhere south of Canal St.	466 (5.0)

a Does not include active-duty New York City firefighters.

**Table 2:** Prevalence of lower and upper respiratory symptoms among the WTC MSP study population (*n* = 9,442).

		Did not report symptoms in year before September 11	
	Reported symptoms in year before September 11 [no. (%)]	New symptoms while working at WTC site [no. (%)]	Symptoms still present in month before exam [no. (%)]
Lower respiratory symptoms			
Dry cough	362 (3.9)	2,541 (28.3)	1,534 (17.1)
Cough with phlegm	325 (3.5)	1,183 (13.1)	742 (8.2)
Shortness of breath	344 (3.7)	1,477 (16.5)	1,266 (14.1)
Wheeze	557 (6.0)	1,232 (14.1)	749 (8.6)
Chest tightness	464 (5.1)	1,258 (14.6)	933 (10.8)
Any lower respiratory symptom	1,451 (15.4)	3,486 (43.8)	2,535 (31.9)
Upper respiratory symptoms			
Sinus-related^a^	2,169 (23.1)	2,219 (30.7)	1,863 (25.8)
Nasal-related^b^	1,967 (20.9)	3,254 (43.8)	2,536 (34.1)
Throat-related^c^	887 (9.4)	3,579 (42.0)	2,450 (28.8)
Any upper respiratory symptom	3,148 (33.5)	3,453 (55.2)	2,772 (44.3)
Any respiratory symptom	3,767 (40.0)	3,443 (61.0)	2,846 (50.4)

a Facial pain or pressure, head or sinus congestion, or postnasal discharge.

b Blowing your nose more than usual, stuffy nose, sneezing, runny nose, or irritation in nose.

c Throat irritation, hoarseness, sore throat, or losing your voice (laryngitis).

**Table 3:** Prevalence of new or worsened respiratory symptoms among WTC workers by date of arrival for work at WTC site and by exposure to the dust cloud (*n* = 9,442).

		Arrived on 11 September					
	All responders (*n* = 9,442) [no. (%)]	In dust cloud (*n* = 1,878) [no. (%)]	Not in dust cloud (*n* = 1,934) [no. (%)]	Arrived 12–13 September (*n* = 2,801) [no. (%)]	Arrived 14–30 September (*n* = 2,133) [no. (%)]	Arrived on or after 1 October (*n*= 666) [no. (%)]	Trend test *p*-value^a^
Lower respiratory symptoms							
Dry cough	2,688 (28.7)	640 (34.2)	587 (30.6)	777 (28.0)	538 (25.5)	140 (21.3)	< 0.001
Cough with phlegm	1,320 (14.1)	328 (17.6)	256 (13.4)	373 (13.5)	275 (13.0)	84 (12.7)	< 0.001
Shortness of breath	1,613 (17.3)	390 (20.9)	298 (15.6)	471 (17.1)	339 (16.1)	109 (16.6)	0.001
Wheeze	1,408 (15.1)	339 (18.3)	296 (15.5)	403 (14.6)	281 (13.4)	85 (13.0)	< 0.001
Chest tightness	1,393 (15.4)	334 (18.5)	268 (14.4)	384 (14.3)	311 (15.2)	91 (14.1)	0.003
Any lower respiratory symptom	4,371 (46.5)	1,017 (54.2)	912 (47.2)	1,232 (44.2)	930 (43.8)	271 (40.8)	< 0.001
Upper respiratory symptoms							
Sinus-related^b^	510 (37.3)	785 (41.9)	712 (36.9)	1,020 (36.6)	783 (37.0)	200 (30.1)	< 0.001
Nasal-related^c^	4,552 (48.4)	982 (52.4)	939 (48.6)	1,334 (47.9)	981 (46.3)	300 (45.1)	< 0.001
Throat-related^d^	4,128 (43.9)	885 (47.2)	847 (43.9)	1,199 (43.1)	923 (43.6)	264 (39.7)	0.001
Any upper respiratory symptom	5,883 (62.5)	1,233 (65.8)	1,205 (62.4)	1,719 (61.7)	1,316 (62.1)	394 (59.2)	0.001
Any respiratory symptom	6,479 (68.8)	1,376 (73.4)	1,345 (69.7)	1,878 (67.3)	1,435 (67.7)	429 (64.5)	< 0.001

a One-sided *p*-values using the Cochran-Armitage trend test.

b Facial pain or pressure, head or sinus congestion, or postnasal discharge.

c Blowing your nose more than usual, stuffy nose, sneezing, runny nose, or irritation in nose.

d Throat irritation, hoarseness, sore throat, or losing your voice (laryngitis).

**Table 4:** Spirometry results (prebronchodilator) among the WTC MSP study population (*n* = 8,384).^a^

		WTC MSP population			
	National population^b^ Never smoker (%)	Never smoker [no. (%)]	Former smoker [no. (%)]	Current smoker [no. (%)]	All [no. (%)]
Normal	87.1	3,396 (73.2)	1,541 (72.8)	1,047 (67.1)	6,031 (71.9)
Obstruction^c^	8.0	237 (5.1)	97 (4.6)	114 (7.3)	451 (5.4)
Low FVC^d^	4.4	940 (20.3)	431 (20.3)	336 (21.5)	1,721 (20.5)
Obstruction and low FVC^e^	0.6	68 (1.5)	49 (2.3)	63 (4.0)	181 (2.2)
Total	NA	4,641 (55.4)	2,118 (25.3)	1,560 (18.6)	8,384 (100.0)

NA, not applicable.

a Only acceptable quality spirometric examinations are included, as described by Miller et al. (2005).

b General U.S. population sample of employed, adult, white males 17–69 years of age who never smoked (Mannino et al. 2003; NHANES III).

c FEV_1_/FVC ratio less than 5th percentile of predicted value and normal FVC.

d FVC less than 5th percentile of predicted value and a normal FEV_1_/FVC ratio.

e FEV_1_/FVC ratio less than 5th percentile of predicted value and FVC less than 5th percentile of predicted value.

**Table 5:** Spirometry results (prebronchodilator) by date of arrival for work at WTC site and exposure to the dust cloud among the WTC MSP study population (*n* = 8,384).^a^

	Arrived on 11 September					
	In dust cloud [no. (%)]	Not in dust cloud [no. (%)]	Arrived 12–13 September [no. (%)]	Arrived 14–30 September [no. (%)]	Arrived on or after 1 October [no. (%)]	Trend test *p*-value^b^
Normal	1,160 (68.5)	1,222 (69.9)	1,781 (71.6)	1,397 (75.3)	453 (78.6)	—
Obstructive^c^	81 (4.8)	96 (5.5)	140 (5.6)	104 (5.6)	28 (4.9)	0.418
Low FVC^d^	408 (24.1)	400 (22.9)	506 (20.3)	318 (17.1)	84 (14.6)	< 0.001
Obstruction and low FVC^e^	44 (2.6)	29 (1.7)	61 (2.5)	36 (1.9)	11 (1.9)	0.095

a Only acceptable quality spirometric examinations are included, as described by Miller et al. (2005).

b One-sided *p*-values using the Cochran-Armitage trend test.

c FEV_1_/FVC ratio less than 5th percentile of predicted value and normal FVC.

d FVC less than 5th percentile of predicted value and a normal FEV_1_/FVC ratio.

e FEV_1_/FVC ratio less than 5th percentile of predicted value and FVC less than 5th percentile of predicted value.
